# What do you do for a living? Toward a more succinct definition of health services research

**DOI:** 10.1186/1472-6963-6-117

**Published:** 2006-09-13

**Authors:** Charles D Phillips

**Affiliations:** 1 Public Health Policy and Management Director, Health Services Research Program, School of Rural Public Health, Texas A&M University System Health Science Center, Texas, USA

## Abstract

This commentary discusses the need for, and the advantages of, a more concise, revised definition of the field of health services research. It argues for a definition that includes not only the topics on which health services research focuses but also the goals of health services research. A number of condensed definitions are provided for consideration.

## Text

Discussing field definitions is usually the stuff of consensus conferences and boards of directors. This task is not usually the province of individual researchers, unless they find some reward in relatively thankless and often futile endeavors. That being said, I am, of course, now about to venture into that daunting area.

I take this rather unusual step for two reasons. First, I have now taught the introductory, core courses in our doctoral program in health services research for a number of years. Like others who teach similar courses, one important part of the initial course meetings is familiarizing students with the various definitions of their chosen field. This task has always left me frustrated. I come away from those sessions thinking that the field of health services research is now well enough established that it deserves a more *mature *statement defining our field than we have heretofore enjoyed. By mature, I mean a statement about the field that has the simplicity and clarity found in the definitions of more long-established fields and disciplines.

Second, with other members of the field in the United States, I participated in a recent meeting and series of exchanges supported by the U.S. Agency for Healthcare Research and Quality concerning the development of competencies for a doctoral degree in health services research. The definition of the field used in that effort was a "close cousin" to what is probably the most widely accepted definition of our field. This is the 2000 AcademyHealth definition of health services research [1]. From that definition, health services research is:

"the multidisciplinary field of scientific investigation that studies how social factors, financing systems, organizational structures and processes, health technologies, and personal behaviors, affect access to health care, the quality and cost of health care, and ultimately our health and well-being. Its research domains are individuals, families, organizations, institutions, communities, and populations."

This definition focuses almost exclusively on defining, as inclusively as possible, the targets of health services research. Such a definitional strategy is well-suited to a "young" field uncertain about both its breadth and the size of the pool of its potential practitioners. It works well for a relatively new field trying to establish itself as a unique entity among older fields of study plowing furrows very near those that we in health services research consider our home turf.

The strategy serves a young field well by casting a wide net for the endeavor. If a researcher is studying something that affects health care or is affected by health care, then that researcher is doing health services research. The pool of practitioners within these broad boundaries then becomes relatively large. The larger this pool becomes, the more likely the young field will be to sustain itself and grow.

Such elaborate boundary setting seems less necessary for more mature disciplines. Look, for example, at economics. As always, definitions are abundant and vary in their complexity. A definition satisfying to most economists might define economics, in the tradition of Lord Robbins from the early 1930s [2], as the investigation of,

"choices made by individuals and societies concerning alternative uses of scarce resources employed to satisfy unlimited wants."

Alternatively, one might consider sociology, which, according to the online version of the Encyclopedia Britannica [3] can be defined as the

"Science of society, social institutions, and social relationships, specifically the systematic study of the development, structure, interactions and collective behavior of organized human groups."

One might want to argue that these academic disciplines have a less applied focus than health services research, making their definitions simpler. However, when one looks at public health, unquestionably an applied science, we discover that public health professionals [4] define their field as activities aimed at

"fulfilling society's interest in assuring conditions in which people can be healthy."

What can those of us in health services research do to arrive at a more "mature" definition that succinctly captures the essence of what we do? I think we can find guidance in conversations that we have all had over the years – that go something like this.

Query: And, what do you do?

Answer: I teach at (work for) X.

Query: What do you teach (do there)?

Answer: Health services research.

Query: What is that?

Unless we feel unusually pedantic or want desperately to immediately end the conversation, we certainly don't recite the Academy definition. Instead, we probably use a definition suggesting that – *Health services research is the study of healthcare costs, quality, or access*.

The use of "or" rather than "and" in that definition may seem troublesome. After all, we would like health care to be effective, equitable, *and *efficient. But, we are still engaging in health services research, even if we look only at effectiveness or investigate access alone. The use of "*or*" clearly allows for such a limited focus and at the same time allows for the possibility that one, should they be so bold, might investigate all three issues simultaneously.

The "holy trinity" of health services research topics (cost, quality, and access) certainly appears in the definition above. Yet, for all of my commitment to brevity, it is too brief. This admirably succinct effort implies, like the current Academy definition, no real goals for our study of these important topics.

In other fields or disciplines, contributing to the discipline or to theory may be the highest goal. Both of these are fine things. But, the roots of health services research rest deeply in the world of applied science somewhere at the intersection of public health and the study of public administration, policy analysis, health administration, community health, and traditional academic disciplines like economics, sociology, and political science [1].

Such a position demands that our applied field state clearly its reason for existence. If we wish to recognize our field's history and solely focus on our goals as a field (or what we do that we believe has high social value), then we might follow the guidance of Lu Ann Aday and her colleagues [5] and respond to the query above with the statement that – *Health services research is the study of how to make healthcare more effective, more equitable, or more efficient*. For those in the field whose bent is more directly toward certain aspects of population health [6], then health services research might be seen simply as a field that – *investigates how and when health services contribute to population health*.

It is important to recognize that the definitions discussed above imply that, for health services researchers, theory development and conceptualization remain secondary concerns. Like many applied disciplines, our field begins with a problem, not a framework. Health services research is concerned, like public health, with population health.

In point of fact, the field can easily be seen as a sub-field of public health. Some health services researchers might find this classification somewhat galling. If so, they would do well to remember that most health services research programs reside in Schools of Public Health and to recognize that those placements were not the result of random chance.

How health services research differs from more general public health research is in its focus on the driving forces behind and the impacts of those institutions and individuals who provide health care. Using the language of public health, health services research spends little of its time on what many consider the heart of general public health, primary prevention. [4] Instead, it focuses largely on secondary and tertiary prevention. Of the two, its heaviest emphasis is on tertiary prevention, understanding how best to provide services to those already ill. Though, just a few years ago, AcademyHealth added an interest group dedicated to the study of *public health systems research*, so the field's representative organization recognizes its role in contributing even to the understanding of primary prevention. [7]

Does the definition of health services research as part of public health mean that those of us in the field can no longer consider ourselves simply health services researchers and that AcademyHealth should close it doors and pack its files? It means nothing of the sort. The umbrella of public health is quite large, and it can accommodate the independence of health services research in the same way that it does environmental health or epidemiology. However, there may be strong political or organizational reasons to eschew the definition of health services research as simply another part of public health and assert its independence as a field. Such reasons can be ignored, but usually one does so at one's peril.

The problem focus of health services research, whether under or beyond the umbrella of public health, does not mean that good health services research is necessarily atheoretical. Instead, it simply means that our core problems or research issues will determine which theoretical or conceptual frameworks, if any, that we use in our work – not the reverse. Of course, all research involves at least some implicit conceptual framework, crude though it may be. But, in health services research that framework can come from the logic of the program under evaluation, common-sense, introspection, or case studies. Some "discipline-approved" set of abstractions need not be involved.

The definitions discussed above also imply that health services research can be done by those who don't really consider themselves health services researchers. The topic (e.g., quality) and the goal of the study (e.g., better quality) determine whether someone is doing health services research. Those of us who consider ourselves health services researchers must recognize that our chosen field is not a closed shop where one needs a union card before they can work. We should also recognize that while many may dabble in our area, the need for scholars whose careers are fully committed to the field is not reduced. In point of fact, those fully committed to health services research will probably, through their teaching or publications, be the ones introducing these "dabblers" to the field.

But, turning back from the implications of these definitions to the definitions themselves, a variety of one-sentence definitions might suffice. For those with less of a passion for brevity, a more traditional health services focus, and desire for a statement of *what we study *as well as *why we study it*, the following might be preferable. Health services research can be seen as – *the study of healthcare costs, quality, or access in order to contribute to population health by making health services more effective, equitable, or efficient*.

One can, of course, argue that health services research, more mature or not, does not "need" a more condensed definition. Some may believe the Academy definition covers the territory well. However, for me, this definition is somewhat defensive and convoluted, burying the core of our field in a flurry of verbiage. As Lohr and Steinwachs [1] recognized in their discussion of the Academy definition in 2002,

"...we need to devise simpler and more effective ways of communicating the content and value of health services research..." (p. 9)

For those, however, who remain satisfied with the current definition, I suggest it is imperative that you add something to that definition defining the goals of our study. One might add text at the beginning of the definition [1] so that health services research becomes

"*the multidisciplinary field of scientific investigation that attempts to improve population health by studying how social......" *(emphasis added for clarity) or

"*the multidisciplinary field of scientific investigation that attempts to improve the effectiveness, equity, or efficiency of healthcare by studying how social......" (*emphasis added for clarity)

Health services researchers are applied researchers who have a mission and a subject. Any useful definition of our field should include both. Adding text like that above to the current definition makes it even more convoluted. However, those who read a revised, long-form definition will now have not only a clear sense of what health services researcher do, these readers will also have a clearer sense of why we study all those things listed in the extended definition.

Should health services researchers be satisfied with one-sentence definitions of our field that are equally adequate for backyard barbeques, idle conversations, or scientific articles? All in all, I think we should be well-satisfied. There are no perfect definitions. There are only useful definitions, and useful definitions don't shroud a field in some specialized vocabulary accessible to only the chosen few. As a field, health services research needs statements that clearly and succinctly communicate to others both the *breadth *and the *importance *of what we do. Those "others" can be our students, scientists working in other parts of our universities or organizations, a staff person working for a member of a public agency, or a staff member at a philanthropic foundation who knows about health but not about health services research.

We also enjoy an additional bonus with a more succinct definition that includes our mission. For our families, friends, and acquaintances, we can finally tell them what we do in a simple, yet arguably orthodox or canonical, fashion. Equally important, we can give them a clear sense of the important social goals to which we have committed our professional lives.

## Pre-publication history

The pre-publication history for this paper can be accessed here:



## References

[B1] Lohr KN, Steinwachs DM (2002). Health services: an evolving definition of the field. HSR.

[B2] Robbins L (1984). An essay on the nature and significance of economic science.

[B3] Sociology. Encyclopedia Britannica. http://concise.britannica.com/article-379009/sociology.

[B4] Institute of Medicine Committee for the Study of the Future of Public Health (1988). The future of public health.

[B5] Kindig DA (1999). Beyond health services research. HSR.

[B6] Aday LA, Begley CE, Lairson DR, Slater CH (1998). Evaluating the healthcare system: Effectiveness, efficiency, and equity.

[B7] Interest Groups. AcademyHealth. http://www.academyhealth.org/interestgroups/index.htm.

